# QTL mapping for young leaf color trait in eggplant (*Solanum melongena* L.) using BSA-seq

**DOI:** 10.3389/fgene.2026.1766303

**Published:** 2026-02-20

**Authors:** Fang Hu, Shaobin Zhang, Chengming Li, Fanchong Yuan, Zhao Song, Kailin Hu, Jiaowen Cheng

**Affiliations:** 1 College of Biology and Agriculture/Guangdong Provincial Key Laboratory of Utilization and Conservation of Food and Medicinal Resources in Northern Region/Guangdong Provincial Engineering and Technology Research Center of Special Fruit and Vegetables in Northern Region, Shaoguan University, Shaoguan, China; 2 College of Horticulture, South China Agricultural University/Key Laboratory of Biology and Genetic Improvement of Horticultural Crops (South China), Ministry of Agriculture and Rural Affairs/Guangdong Vegetables Engineering Research Center, Guangzhou, China; 3 Vegetable Research Institute, Guangdong Academy of Agricultural Sciences, Guangzhou, China

**Keywords:** BSA-seq, eggplant, genetic analysis, QTL, young leaf color

## Abstract

Young leaf color is a crucial agronomic trait in eggplant (*Solanum melongena* L.) significantly influencing photosynthetic efficiency, stress resistance, fruit quality, and ornamental value. However, research focusing on this trait remains relatively scarce. In this study, an F_2_ (EP02×EP01) segregating population (n = 646) was developed from a cross between the purple young-leaved line EP02 and green young-leaved line EP01. Phenotypic characterization of the F_2_ population revealed that the segregation ratio of green, purple-green, and purple phenotypes conformed to the expected 1:2:1 Mendelian ratio (χ^2^ = 3.40, P > 0.05), indicating that the young leaf color trait in eggplant is controlled by a single incompletely dominant gene. To identify candidate loci associated with young leaf color, 30 individuals with extreme purple phenotypes and 30 individuals with extreme green phenotypes were selected from the F_2_ population to establish two DNA bulks, namely, the purple trait bulk (ZS-pool) and the green trait bulk (LS-pool), respectively. Bulked Segregant Analysis (BSA) combined with whole-genome resequencing was performed on the two parental lines (EP02 and EP01) and the two bulks (ZS-pool and LS-pool). A total of 1,416,609 high-quality single nucleotide polymorphisms (SNPs) were generated and used for quantitative trait locus (QTL) mapping. Using the SNP-index method and Euclidean Distance (ED) analysis, a single QTL associated with young leaf color was identified on chromosome 10, covering a physical interval of 17.49 Mb (59,315,357-76,806,837 bp). Integrated analysis of SNP indices, ED values, and gene functional annotations suggested that *Smechr1002213*, *Smechr1001752*, and *Smechr1001815* might be the candidate gene regulating young leaf color formation in eggplant. The findings of this study will lay a foundation for gene isolation and the elucidation of the genetic mechanisms underlying young leaf coloration in eggplant.

## 1 Introduction

Eggplant (*Solanum melongena* L.), an economically important member of the Solanaceae family, has a long cultivation history and is widely grown throughout Southeast Asian, African, and the Mediterranean region (Kalloo and Bergh, 1993; Knapp et al., 2013). As one of the most widely consumed vegetable crops globally, eggplant holds significant economic value within agricultural production systems. According to statistics from the Food and Agriculture Organization of the United Nations (FAO, 2022) in 2022, the global annual production of eggplant exceeds 58 million tons, with China being the world’s largest producer, accounting for approximately 65% of the global total output. This prominent economic status underscores the necessity of advancing genetic improvement research on eggplant to meet evolving market demands and enhance agricultural sustainability. In recent years, the purple-leaf trait of eggplant has garnered increasing research attention, driven by rising consumer demand for high-quality vegetables and the rapid development of the ornamental horticulture sector (Zhang et al., 2014a; Zhang et al., 2016).

Leaf coloration is determined by the synergistic interaction of pigments, including chlorophyll, carotenoids, and anthocyanins. As the core pigment involved in photosynthesis, Chlorophyll directly regulates the light-capturing efficiency and photosynthetic productivity of leaves through its content and composition. Anthocyanin, as secondary metabolites, are mainly accumulated in the vacuoles of epidermal or palisade tissues of leaves and widely distributed in the stems, leaves, flowers, and fruits of plants (Cammareri et al., 2024). Under stress conditions, anthocyanin not only act as an “optical filter” by absorbing excess visible light (especially green light) and ultraviolet radiation to reduce photoinhibition and photooxidative damage, thereby protecting the photosynthetic system, but also exhibit antioxidant activities—alleviating cell dehydration and membrane damage caused by stresses such as low temperature and drought (Mendez et al., 1999). Additionally, they can rapidly scavenge reactive oxygen species (ROS) bursts triggered by pathogen infection or pest infestation, thereby reducing oxidative stress damage in cells (Grotewold, 2006; Harborne and Williams, 1992).

The purple color of eggplant leaves primarily originates from the accumulation of anthocyanins in vacuoles (Cammareri et al., 2024). Anthocyanins endow eggplant leaves with color gradients ranging from deep purple to light purple, which not only enhance the inherent visual characteristics of the crop but also enrich the color combinations in landscape designs (Zhang et al., 2014b). Purple-leaf eggplant integrates ornamental and edible functions, adapts to diverse cultivation scenarios such as potted cultivation, balcony planting, and agritourism, and aligns with the modern family gardening pursuit of both functionality and aesthetics.

Anthocyanin accumulation has long been regarded as a key trait for quantitative trait research in eggplant (Nunome et al., 2001). Numerous studies have extensively investigated anthocyanin accumulation in various tissues and organs of eggplant, performing quantitative trait locus (QTL) mapping through genetic map construction (Barchi et al., 2012; Wei et al., 2020; Guan et al., 2022). These studies revealed that anthocyanin accumulation is primarily controlled by QTLs distributed across chromosomes 1, 2, 5, 6, 7, 8, 10, 11, and 12 (Barchi et al., 2012; Cericola et al., 2014). The QTLs with the greatest contribution to genetic variation, particularly those responsible for tissue-specific anthocyanin accumulation, are predominantly concentrated in specific regions of chromosomes 5 and 10 (Frary et al., 2014; Wei et al., 2020). Among them, the region on chromosome five is mainly associated with anthocyanin presence in vegetative tissues, such as hypocotyls, stems, leaf veins, and pedicels, while the region on chromosome 10 is mainly related to anthocyanin accumulation in stems, leaves, leaf veins, floral organs, and fruits (Cammareri et al., 2024).

Genes encoding anthocyanin biosynthetic enzymes, including phenylalanine ammonia-lyase (PAL) (Huang et al., 2010), chalcone synthase (CHS) (Glagoleva et al., 2019), chalcone isomerase (CHI) (Sun et al., 2019), dihydroflavonol 4-reductase (DFR) (Nakatsuka et al., 2003), anthocyanin synthase (ANS) (Liu et al., 2013), and flavonoid 3-O-glucosyltransferase (UFGT) (Fukuchi-Mizutani et al., 2003), as well as key regulatory transcription factors such as MYB family members, bHLH family members, and WD40 transcription factors (Albert et al., 2014; Pattanaik et al., 2014; Cammareri et al., 2024), have been extensively characterized in various plant species. Currently, key genes regulating anthocyanin synthesis in eggplant fruits, such as *SmMYB1*, *SmANS*, and *SmPAL*, have been identified (Zhou et al., 2021; Zhou X. et al., 2022; You et al., 2023), and research on their regulatory mechanisms has also made considerable progress.

Despite significant progress in elucidating the anthocyanin biosynthesis pathway and identifying QTLs in eggplant, most existing studies have primarily focused on fruit pigmentation. To date, genetic mapping of anthocyanin accumulation in leaves—particularly for the young leaf color trait—and the associated identification of candidate genes remain unreported. To address this gap, we first constructed a large F_2_ segregating population (n = 646) derived from parental lines with green and purple young leaves, followed by phenotypic investigation and genetic analysis of the young leaf color trait in eggplant. Subsequently, we established extreme phenotype pools for young leaf color and employed the bulked segregant analysis (BSA) approach to map the genetic loci governing this trait. Finally, by integrating the SNP-index and ED values generated from gene mapping with gene functional annotations, we preliminarily screened out potential candidate genes regulating young leaf color formation in eggplant. The findings of this study will lay a foundation for gene isolation and the elucidation of the genetic mechanisms underlying young leaf coloration in eggplant.

## 2 Materials and methods

### 2.1 Plant materials

The purple young leaf line EP02 and the green young leaf inbred line EP01, both developed by successive generations of self-pollination at the Key Laboratory of Biology and Genetic Improvement of Horticultural Crops (South China), Ministry of Agriculture and Rural Affairs, were used as the female and male parents, respectively. The F_1_ generation (EP02×EP01) was obtained via artificial hybridization, followed by strict self-pollination of F_1_ plants to establish the F_2_ segregation population (EP02×EP01). All plants from the EP01 (n = 30), EP02 (n = 30), F_1_ (n = 30), and F_2_ (n = 646) generations were cultivated at the Main Campus Teaching & Research Base of South China Agricultural University (SCAU) in Guangzhou, China (23°N, 113°E) during the spring of 2021 under standard agronomic practices, and were used to analyze the distribution and inheritance patterns of eggplant young leaf color. All plants used in this study were grown in an open field.

### 2.2 Phenotyping

**FIGURE 1:**
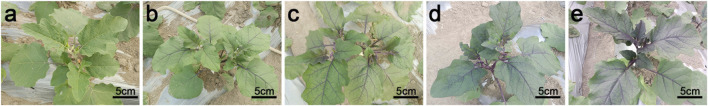
The phenotype of eggplant leaf color. **(a)** represents the green phenotype consistent with that of EP01; **(b–d)** denote the young leaf phenotypes with varying degrees of purple coloration intermediate between the two parental lines, which are collectively designated as the Purple‐green type; **(e)** represents the purple phenotype consistent with that of EP02.

After primary floral buds emerged in the EP02, EP01, F_1_, and F_2_ populations, the anthocyanin accumulation trait of the adaxial leaf lamina—an indicator of young leaf color—was evaluated on newly expanded leaves from the upper canopy. Phenotypes of individual plants in EP01, EP02, F_1_ and F_2_ populations were recorded, while phenotypes in the F_2_ population were counted and a frequency distribution histogram was generated. Based on field phenotypic observations, the young leaf color of eggplant was classified into three types: green (Green, same as the phenotype of EP01), purple (Purple, same as the phenotype of EP02), and an intermediate phenotype termed purple-green (Purple-green) (Figure 1). Phenotypic evaluation was performed independently by two observers to ensure the accuracy of phenotype recording, and inconsistent evaluations were verified by re-observation in the field.

### 2.3 DNA extraction, bulk construction and re-sequencing

Genomic DNA was extracted from both the parental pools and the samples used for constructing the extreme trait pools using a modified CTAB method. The parental pools (EP02-pool and EP01-pool) were constructed by pooling juvenile leaves from ten uniformly sized plants of each inbred line (EP02 and EP01). Two extreme trait pools were prepared by mixing equal amounts of genomic DNA from 30 F_2_ individuals with purple young leaves and 30 F_2_ individuals with green young leaves, designated as the ZS-pool and LS-pool, respectively.

A total of four whole-genome resequencing DNA libraries were prepared, corresponding to the two extreme trait bulks (ZS-pool, and LS-pool) and the two parental pools (EP02-pool and EP01-pool), respectively. Qualified DNA samples were randomly fragmented using a Covaris instrument, and fragments of approximately 500 bp in length were obtained after fragment size selection. The selected fragments were subjected to end repair and 3′-end adenylation, followed by ligation of library adapters to both ends. The adapter-ligated libraries were subjected to linear amplification (LM-PCR). An appropriate amount of the amplified products was subjected to single-strand separation and circularization. The circularized libraries were converted into DNA nanoballs via rolling circle amplification (RCA), and sequenced on the BGISEQ-500 platform (BGI, Shenzhen, China) after passing quality control.

Raw sequencing reads were subjected to quality control and filtration using Soapnuke software, following a stringent filtering pipeline: (1) removal of reads containing adapter sequences; (2) exclusion of low-quality reads, defined as those with more than 40% of bases exhibiting a Phred quality score ≤20; and (3) discarding of reads containing over 5% of ambiguous bases (N). This procedure yielded high-quality clean reads suitable for subsequent analysis. The clean reads from each sample were independently aligned to the eggplant reference genome (“01.SME-HQ-reference.fasta”) (Barchi et al., 2019) using BWA (version 0.7.15-r1140) with default parameters. The resulting Sequence Alignment/Map (SAM) files were then converted to sorted Binary Alignment/Map (BAM) files using SAMtools (version 1.9). To ensure the reliability of subsequent variant calling, only reads with a mapping quality (mapQ) value greater than 10 and properly paired alignments were retained for further analysis.

### 2.4 BSA-seq

BSA analysis was performed based on high-quality SNPs identified from the EP02, EP01, ZS-pool, and LS-pool. SNP calling and filtration were conducted using GATK (version 4.1.2) to obtain highly reliable population-wide variant data, which were subsequently aligned to the reference genome. To ensure the robustness of subsequent analyses, variants with abnormal values of the following metrics were excluded: sequencing depth (DP < 60 or DP > 1000), variant quality (QUAL <50.0, MQ < 50.0, QD < 2.0), Fisher’s exact test strand bias (FS > 40.0), allele number (AN < 5), and Strand Odds Ratio (SOR >5.0). This stringent filtration procedure was designed to eliminate false-positive variants arising from sequencing or alignment errors. For each SNP locus, the fourth power of Euclidean Distance (ED^4^) was calculated between the two extreme pools using the following formula:

$${ED}^{4}={\left(\frac{{Alt}_{ls}}{{Depth}_{ls}}-\frac{{Alt}_{zs}}{{Depth}_{zs}}\right)}^{4}$$

Where Alt_ls_ and Alt_zs_ represent the read counts of the alternative allele in LS-pool, alternative allele in ZS-pool. Depth_ls_ and Depth_zs_ represent the total read depth in the respective pool. The top 1% of ED^4^ values across the genome was defined as the significance threshold. Genomic regions where the LOESS-fitted ED^4^ values exceeded this threshold were identified as candidate intervals associated with the regulation of young leaf color.

​The ΔSNP-index, which served as the measure of association, was derived by calculating the difference in SNP-index values between the two bulk pools. The ΔSNP-index was computed as:

$$\Delta SNP-index=\frac{{lsN}_{alt}}{{lsN}_{alt}+N_{ref}}-\frac{{zsN}_{alt}}{{zsN}_{alt}+N_{ref}}$$

where lsN_alt_, zsN_alt_ and N_ref_ denote the read counts of the alternative allele in LS-pool, alternative allele in ZS-pool and reference alleles, respectively. This computation was performed for all qualified SNPs using sliding window analysis with a window size of 1 Mb and a step size of 500 kb. A 95% confidence interval was applied to the fitted values, with values exceeding this interval used to delineate significant QTL regions.

Finally, the candidate intervals detected by the ED^4^ and ΔSNP-index methods were overlapped to define a consensus genomic interval as the key QTL responsible for young leaf color variation in eggplant.

## 3 Results

### 3.1 Inheritance of the eggplant young leaf color

**FIGURE 2:**
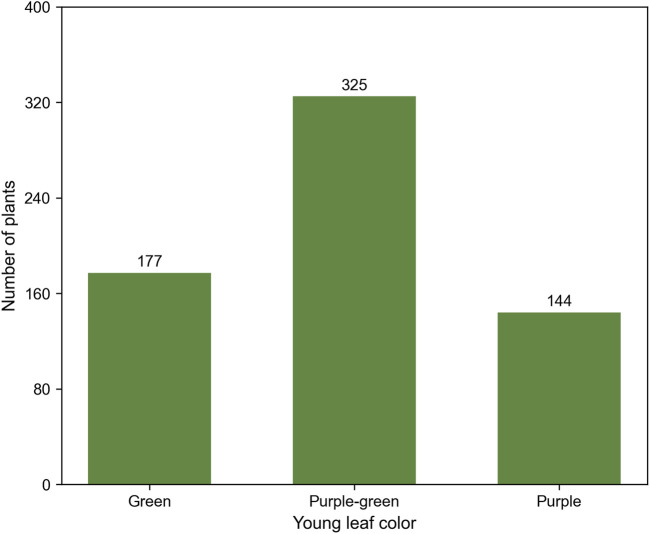
Frequency distribution of young leaf color in the F_2_ population.

The results showed that all EP02 plants exhibited purple young leaves (Purple), all EP01 plants exhibited green young leaves (Green), and all F_1_ plants displayed an intermediate phenotype between the two parents (Figure 1). In the F_2_ population, 177 plants showed green young leaves (Green), similar to those of EP01, 144 plants displayed purple young leaves (Purple), similar to those of EP02, and 325 plants an intermediate phenotype between the two parents, with varying degrees of purple coloration, which was uniformly designated as Purple-green (Figure 2). The observed Green/Purple-green/Purple segregation fit the expected 1:2:1 ratio (χ2 = 3.40, P > 0.05) (Table 1), indicating that the young leaf color trait in eggplant is controlled by a single incompletely dominant gene.

**TABLE 1:** Field-based phenotypic statistics of plants.

Generation	No. of green plants	No. of purple-green plants	No. of purple plants	Expected ratio	χ^2^	P
EP02	0	0	30	​	​	​
EP01	30	0	0	​	​	​
EP02 × EP01 F_1_	0	30	0	​	​	​
EP02 × EP01 F_2_	177	325	144	1:2:1	3.40	0.18

### 3.2 Sequencing data and variant statistics

Whole-genome resequencing of the two parental lines (EP02, EP01) and the two extreme phenotype pools bulks (ZS-pool, LS-pool) generated a total of 238.83 GB of high-quality clean data following bioinformatic processing. All libraries exhibited high mapping efficiency, with an average alignment rate of 99.58% to the reference genome (“01.SME-HQ-reference.fasta”). The average sequencing depth across samples ranged from 43.57× to 47.26×. Additional quality metrics, including GC content, Q20 (95.59%), and Q30 (89.87%), were all within the expected ranges for robust whole-genome sequencing data (Table 2). These high-quality sequencing data ensure the accuracy and reliability of subsequent variant calling and BSA analysis.

**TABLE 2:** Summary of genome resequencing data of eggplant young leaf color traits.

Sample	Total reads	Base number (Gb)	Mapped rate (%)	Q20 (%)	Q30 (%)	GC content (%)	Average depth (X)
EP02-pool	404,430,780	60.66	99.77	95.49	89.65	37.37	45.68
EP01-pool	381,166,916	57.18	99.21	95.78	90.22	36.99	43.57
ZS- pool	413,745,076	62.06	99.65	95.63	90	35.94	47.26
LS- pool	392,879,342	58.93	99.69	95.44	89.61	36.74	44.42
Average	398,055,529	59.71	99.58	95.59	89.87	36.76	45.23

The EP02-pool and EP01-pool represent the parental pools, while the ZS-pool and LS-pool correspond to the purple young leaves and green young leaves extreme pools, respectively.

### 3.3 Identification of loci for young leaf coloration in eggplant via BSA

**FIGURE 3:**
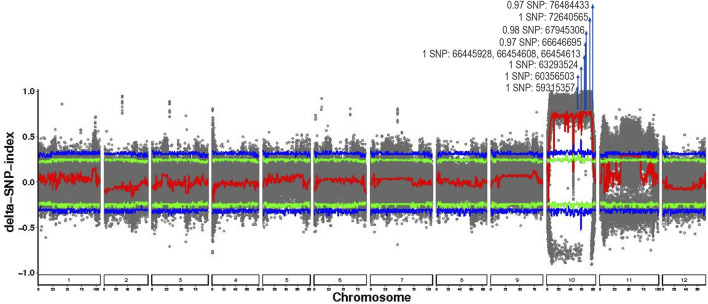
Plot of genome-wide ΔSNP-index for young leaf color of eggplant. Distribution of Δ(SNP-index) across all chromosomes. The fitted line is shown in red, significance thresholds at 95% and 99% levels are indicated by green and blue lines, respectively. The part marked by the arrow indicates the QTL peak and candidate SNPs.

**FIGURE 4:**
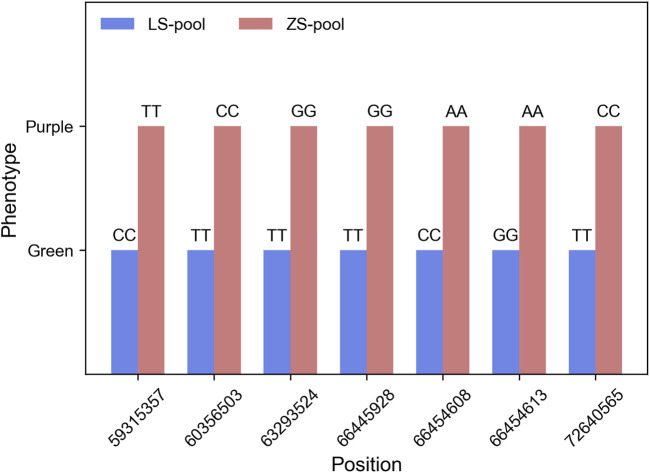
The genotype of SNP sites in ZS-pool and LS-pool when the Δ(SNP-index) value is 1.

Candidate intervals for young leaf color were identified using both the SNP-index and ED analysis methods. Following the filtration of parentally segregated variants, 1,416,609 high-quality SNPs were obtained for analysis. The Δ(SNP-index) was calculated as the allele frequency difference between the ZS-pool and LS-pool. At the 95% confidence level, a distinct Δ(SNP-index) peak was observed on chromosome 10 (Figure 3), with values reaching one in the core interval (Table 3; Figure 4), which is indicative of tight genetic linkage between this interval and the target young leaf color trait.

**TABLE 3:** High-confidence loci and candidate genes controlling leaf color in eggplant.

Position	ΔSNP-index	ED^4^	REF	ALT	Type	Gene_ID	Homologous species	Homologous protein
59315357	1	3.998	C	T	INTRON	Smechr1001434	*Solanum tuberosum*	E3 ubiquitin-protein ligase SHPRH isoform X1
60356503	1	3.998	C	T	INTRON	Smechr1001462	*Solanum tuberosum*	2-Hydroxy-6-oxononadienedioate/2-hydroxy-6-oxononatrienedioate hydrolase isoform X2
63293524	1	3.998	T	G	INTRON	Smechr1001567	*Solanum pennellii*	Alpha-amylase 3, chloroplastic
66445928	1	3.998	T	G	INTRON	Smechr1001745	*Capsicum annuum*	Hypothetical protein T459_07512
66454608	1	3.998	A	C	INTRON	Smechr1001747	*Solanum chilense*	Hypothetical protein EJD97_018690
66454613	1	3.998	A	G	INTRON	Smechr1001747	*Solanum chilense*	Hypothetical protein EJD97_018690
72640565	1	3.998	C	T	DOWNSTREAM	Smechr1002106	*Solanum pennellii*	Uncharacterized protein LOC107018466
66446035	0.99	3.765	C	A	INTRON	Smechr1001745	*Capsicum annuum*	Hypothetical protein T459_07512
66446013	0.98	3.755	C	G	INTRON	Smechr1001745	*Capsicum annuum*	Hypothetical protein T459_07512
63292123	0.98	3.744	A	G	UPSTREAM	Smechr1001567	*Solanum pennellii*	Alpha-amylase 3, chloroplastic
65672976	0.98	3.733	A	G	INTRON	Smechr1001691	*Solanum pennellii*	GDSL esterase/lipase CPRD49-like isoform X2
64429319	0.98	3.712	G	C	UTR_3_PRIME	Smechr1001617	*Solanum tuberosum*	Uncharacterized protein LOC102604964 isoform X1
65673004	0.98	3.712	T	C	INTRON	Smechr1001691	*Solanum pennellii*	GDSL esterase/lipase CPRD49-like isoform X2
**67945306**	**0.98**	3.69	**A**	**G**	**DOWNSTREAM**	**Smechr1001815**	***Solanum tuberosum***	**RING finger and transmembrane domain-containing protein 2**
69549588	0.98	3.68	A	G	DOWNSTREAM	Smechr1001899	*Solanum tuberosum*	ACT domain-containing protein ACR4 isoform X2
76806837	0.98	3.68	A	G	EXON:NON_SYNONYMOUS_CODING	Smechr1002232	*Solanum demissum*	Hypothetical protein SDM1_27t00022
64429303	0.98	3.669	C	T	UTR_3_PRIME	Smechr1001617	*Solanum tuberosum*	Uncharacterized protein LOC102604964 isoform X1
69549574	0.98	3.669	A	T	DOWNSTREAM	Smechr1001899	*Solanum tuberosum*	ACT domain-containing protein ACR4 isoform X2
63314887	0.98	3.648	G	T	EXON:NON_SYNONYMOUS_CODING	Smechr1001569	*Solanum tuberosum*	Uncharacterized protein LOC102599958
64429262	0.98	3.637	G	A	UTR_3_PRIME	Smechr1001617	*Solanum tuberosum*	Uncharacterized protein LOC102604964 isoform X1
**66646695**	**0.97**	**3.616**	**G**	**A**	**INTRON**	**Smechr1001752**	***Solanum pennellii***	**Solute carrier family 40 member 2-like**
66455205	0.97	3.574	A	C	INTRON	Smechr1001747	*Solanum chilense*	Hypothetical protein EJD97_018690
**76484433**	**0.97**	**3.574**	**C**	**G**	**DOWNSTREAM**	**Smechr1002213**	***Solanum melongena***	**Myb1**
64504935	0.97	3.533	G	C	DOWNSTREAM	Smechr1001620	*Solanum tuberosum*	Short-chain dehydrogenase TIC 32, chloroplastic-like
64533133	0.97	3.533	G	T	INTRON	Smechr1001621	*Solanum tuberosum*	THO complex subunit 4A-like
63323387	0.97	3.523	A	G	UPSTREAM	Smechr1001570	*Solanum tuberosum*	Single-stranded DNA-binding protein WHY1, chloroplastic
64422868	0.97	3.512	T	C	INTRON	Smechr1001617	*Solanum tuberosum*	Uncharacterized protein LOC102604964 isoform X1
66297789	0.97	3.512	C	T	DOWNSTREAM	Smechr1001734	*Solanum pennellii*	Putative disease resistance protein At4g10780
66454666	0.97	3.492	G	A	INTRON	Smechr1001747	*Solanum chilense*	Hypothetical protein EJD97_018690
66612101	0.97	3.492	T	C	UPSTREAM	Smechr1001751	*Solanum tuberosum*	F-box/LRR-repeat protein 3
76123767	0.97	3.492	C	T	INTRON	Smechr1002207	*Solanum tuberosum*	Uncharacterized protein LOC102588539
63314996	0.97	3.472	G	A	EXON:SYNONYMOUS_CODING	Smechr1001569	*Solanum tuberosum*	Uncharacterized protein LOC102599958
63323404	0.96	3.472	G	T	UPSTREAM	Smechr1001570	*Solanum tuberosum*	Single-stranded DNA-binding protein WHY1, chloroplastic
64411992	0.97	3.472	T	C	DOWNSTREAM	Smechr1001616	*Nicotiana tomentosiformis*	Ras-related protein RABE1a-like

The bold-marked genes are candidate genes potentially controlling young leaf color in eggplant. REF: reference allele, ALT: alternate allele.

**FIGURE 5:**
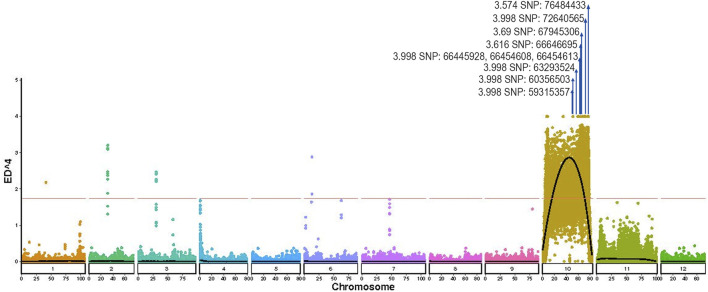
Scatter diagram of ED^4. The black line indicates the fitted ED^4 value, and the red dotted line indicates the significance association threshold (1.74). The part marked by the arrow indicates the QTL peak and candidate SNPs.

Independent ED analysis also detected a significant peak in the identical chromosomal region (Figure 5), where ED values surpassed the genome-wide 95% confidence threshold and approached a maximum of 3.998 (Table 3; Figure 5). The black fitted curve in Figure 5 further defined the peak boundaries, showing perfect concordance with the Δ(SNP-index) signal. By intersecting the candidate intervals identified by these two mutually validated methods, a QTL for young leaf color was fine-mapped to a 17.49 Mb region on chromosome 10 (59,315,357-76,806,837 bp) (Table 3).

Combined analysis of SNP indices, ED values, and gene functional annotations suggested that *Smechr1002213* (a MYB1 transcription factor that activates anthocyanin biosynthesis), *Smechr1001752* (a solute carrier family 40 member 2-like protein involved in metal ion transport), and *Smechr1001815* (a RING finger and transmembrane domain-containing protein associated with protein ubiquitination) might be the candidate gene regulating young leaf color formation in eggplant (Table 2), which was designated as *adlan10.1*.

## 4 Discussion

Young leaf color is a pivotal agronomic trait integrating ornamental value, stress resistance, and photosynthetic efficiency in eggplant (Grotewold, 2006; Harborne and Williams, 1992), yet its genetic basis remains poorly understood compared to that of fruit pigmentation. In this study, a large F_2_ segregating population and BSA-seq technology were employed to investigate the genetic rules governing young leaf color in eggplant, delineate the initial mapping interval, and preliminarily screen candidate genes. These findings lay a foundation for the cloning of genes related to young leaf color and the elucidation of their underlying genetic regulatory mechanisms in eggplant.

Genetic analysis revealed that eggplant young leaf color trait was controlled by a single incompletely dominant gene. This finding diverges from previous reports, which indicated that anthocyanin accumulation in the adaxial leaf lamina (adlan) is governed by polygenic inheritance with a major gene playing a significant role, exhibiting a heritability of 0.93 (Barchi et al., 2012). Further genome-wide association analysis by Cericola et al. (2014) identified strong associations between loci E10.2 and E10.3 on chromosome E10 and anthocyanin pigmentation on both adlan and abaxial leaf lamina anthocyanin, supporting a polygenic interaction model. In a comprehensive review, Cammareri et al. (2024) elaborated on the complexity of leaf anthocyanin inheritance, highlighting that the E10.1 gene cluster primarily regulates anthocyanin accumulation in the adlan, whereas the E10.2 and E10.3 clusters coordinately control pigmentation in veins and the abaxial leaf lamina. The discrepancies observed in these genetic regulatory patterns may stem from trait specificity, such as differences between young and mature leaves. This tissue-specific regulatory mechanism provides a plausible explanation for the variations in genetic models reported across different studies. Moreover, in tomato, leaf anthocyanin accumulation is controlled by multiple interacting genes, including Anthocyanin fruit (*Aft*), Aubergine (*Abg*), and atv (Anthocyanin without veining), which exhibit additive or epistatic effects (Povero et al., 2011). This interspecific difference further indicates the complexity and specificity of anthocyanin regulatory networks in Solanaceae plants.

Leveraging its inherent advantages of high resolution and accuracy, BSA-Seq technology has been widely applied in gene mapping studies for various traits in Solanaceae crops, including the identification of genes associated with orange fruit color and exserted stigma sterility in tomato (Cheng et al., 2021; Zhou M. et al., 2022), as well as the gene mapping of leaf and fruit color traits in pepper (Sun et al., 2024; Feng et al., 2024). In this study, a total of 1,416,609 high-confidence SNPs were identified using BSA-Seq technology. Subsequently, by integrating the SNP-index method and Euclidean distance (ED) analysis, a QTL interval spanning 17.49 Mb was mapped on chromosome 10 of eggplant, with a physical position ranging from 59,315,357 to 76,806,837 bp (Table 3). The highly consistent signals obtained from these two complementary analytical approaches not only enhanced the reliability of this QTL interval and reduced the risk of false positives but also laid a solid foundation for subsequent gene identification and functional validation.

Previous studies have reported that multiple QTLs associated with purple leaves in eggplant are all located on chromosome 10, including *adlanE10.ML,MT, adlan*, *adlan10.1BT,ML,MT*, *lla10.1*, *ablanE10a.m.L*, and *ablanE10.MT* (Table 4). Among these loci, *adlan10.1BT,ML,MT* is physically located at 95 Mb, showing a significant difference from the adlan10.1 locus identified in this study. Due to the lack of available physical position information, comparative analysis could not be performed for the other loci. In addition, the adlan10.1 locus reported here is also distinct from the QTLs regulating stem color, flower color, fruit color, and vein patterning in eggplant (Cammareri et al., 2024). Given that the genetic regulatory patterns of the aforementioned traits are different from those of the young leaf color trait focused on in this study, it is inferred that the core regulatory genes governing these traits may be distinct, which further reflects the tissue-specific regulation of anthocyanin accumulation in eggplant.

**TABLE 4:** Anthocyanin QTLs and candidate genes.

QTL name	Position (cM)	Position (Mb)	Candidate gene	References
***adlan10.1***	​	**59.31-76.81**	***Smechr1002213,Smechr1001752,Smechr1001815***	​
*adlanE10.ML,MT*	69.39	​	*3 GT,AN1,ANT2*	Barchi et al. (2012)
*adlan*	69.13	​	​	Cericola et al. (2014)
*adlan*	69.39	​	​	Cericola et al. (2014)
*adlan10.1BT,ML,MT*	236.98	95	*peroxidase,PDI*	Toppino et al. (2020)
*lla10.1*	109.8	​	​	Frary et al. (2014)
*ablanE10a.m.L*	68.92	​	*3 GT,AN1,ANT2*	Barchi et al. (2012)
*ablanE10.MT*	68.58	​	*3 GT,AN1,ANT2*	Barchi et al. (2012)

All the above QTLs, are located on chromosome 10. The QTL, identified in this study is highlighted in bold. adlan: adaxial leaf lamina anthocyanin; ablan: abaxial leaf lamina anthocyanin; lla: Anthocyanin content of leaf laminae.

Within the candidate interval, *Smechr1002213*, *Smechr1001752*, and *Smechr1001815* were identified as potential candidate genes regulating pigment formation in young eggplant leaves. Functional annotation revealed that *Smechr1002213* shares homology with *Myb1*, a well-characterized transcription factor known to activate anthocyanin biosynthesis in Solanaceae crops. *SmMYB1* has been shown to regulate fruit anthocyanin accumulation in eggplant by binding to the promoters of key structural genes such as *SmDFR* and *SmANS* (Zhang et al., 2014b). Furthermore, in potato, *StAN1* (a MYB homolog) coordinately regulates anthocyanin biosynthesis with *bHLH* partners, and its expression is modulated by light and sucrose signals, creating a direct link between photosynthetic sugar status and pigmentation (Liu et al., 2015). Based on these findings, it is hypothesized that *Smechr1002213* may play a similar regulatory role in young leaf anthocyanin accumulation in eggplant, which warrants further functional verification. *Smechr1001752* (SLC40A2-like), as a metal ion transporter, may enhance pigment stability by modulating the intracellular concentrations of ions such as iron and magnesium, thereby affecting the activity of anthocyanin modification enzymes and the formation of anthocyanin-metal chelates (Gonzalez et al., 2008). Notably, metal ions also serve as key cofactors in chlorophyll synthesis and the photosynthetic electron transport chain. Thus, this gene may mediate the balance between anthocyanin accumulation and photosynthetic efficiency. *Smechr1001815* is homologous to a RING finger and transmembrane domain-containing protein from *Solanum tuberosum*. Proteins harboring RING finger domains often function as E3 ubiquitin ligases, which are involved in protein ubiquitination modification. In *Arabidopsis*, RING-type E3 ligases have been shown to influence anthocyanin synthesis by regulating the stability of *PAP1*/*MYB75* (Maier et al., 2013). However, all of the above are candidate genes, and further functional validation is still required.

## 5 Conclusion

This study clarified that the young leaf color trait of eggplant is controlled by a single incompletely dominant gene. Using BSA-seq, we identified a QTL interval on chromosome 10 spanning 17.49 Mb (59,315,357-76,806,837 bp). Integrated analysis of SNP-index, ED values, and gene functional annotations suggested that *Smechr1002213* (a MYB1 transcription factor that activates anthocyanin biosynthesis), *Smechr1001752* (a solute carrier family 40 member 2-like protein involved in metal ion transport), and *Smechr1001815* (a RING finger and transmembrane domain-containing protein associated with protein ubiquitination) might be the candidate gene regulating young leaf color formation in eggplant. These findings lay a solid foundation for the isolation of key genes and the elucidation of the genetic mechanisms underlying young leaf coloration in eggplant, and provide a theoretical basis for the genetic improvement of eggplant with integrated ornamental and agronomic traits.

## Data Availability

The original contributions presented in the study are included in the article/supplementary material, further inquiries can be directed to the corresponding author.
